# Supplementary material for: Associations between Milk Fatty Acid Profile and Body Condition Score, Ultrasound Hepatic Measurements and Blood Metabolites in Holstein Cows
Source: Animals (Basel). 2022 May 6;12(9):1202. doi: 10.3390/ani12091202 (PMC9104722; doi:10.3390/ani12091202)
Supplement: Supplementary file 1 [file animals-12-01202-s001.zip › animals-1665159-supplementary.pdf]

Supplementary Materials

Table S1. Descriptive statistics for milk production traits and individual fatty acids (n = 297).

Traits [1]	Common Name	Mean	SD	P1 [2]	P99 [2]
Milk yield, kg		37.86	8.31	20.54	59.03
Milk composition, kg					
Fat		3.55	0.71	1.78	5.36
Protein		3.22	0.41	2.59	3.88
Individual fatty acids, g/100 g of total					
SFA					
4:0	Butyric acid	1.66	0.31	1.09	2.42
6:0	Caproic acid	1.66	0.28	1.03	2.47
8:0	Caprylic acid	1.25	0.20	0.74	1.75
10:0	Capric acid	2.97	0.57	1.45	4.06
11:0	Undecanoic acid	0.11	0.05	0.02	0.23
12:0	Lauric acid	3.55	0.72	1.66	4.93
13:0 iso	iso Tridecanoic acid	0.03	0.01	0.01	0.05
13:0	Tridecanoic acid	0.24	0.08	0.08	0.42
14:0 iso	iso Tetradecanoic acid	0.12	0.03	0.05	0.19
14:0	Myristic acid	11.02	1.37	7.47	13.66
15:0 iso	iso Pentadecanoic acid	0.20	0.04	0.12	0.29
15:0 ante	ante Pentadecanoic acid	0.41	0.06	0.24	0.54
15:0	Pentadecanoic acid	1.28	0.30	0.62	1.89
16:0 iso	iso Hesadecanoic acid	0.25	0.06	0.16	0.43
16:0	Palmitic acid	30.62	2.73	25.66	38.03
17:0 iso	iso Heptadecanoic acid	0.33	0.04	0.24	0.45
17:0 ante	ante Heptadecanoic acid	0.43	0.08	0.30	0.63
17:0	Margaric acid	0.57	0.09	0.41	0.84
18:0	Stearic acid	10.93	2.18	6.71	16.81
20:0	Arachidic acid	0.18	0.04	0.10	0.29
22:0	Behenic acid	0.06	0.02	0.03	0.10
24:0	Lignoceric acid	0.04	0.01	0.02	0.08
MUFA					
10:1 (c9)	Caproleic acid	0.28	0.07	0.07	0.42
14:1 (c9)	Myristoleic acid	0.84	0.24	0.33	1.40
16:1 (t9)	Palmitelaidic acid	0.20	0.04	0.13	0.29
16:1 (c9)	Palmitoleic acid	1.44	0.36	0.72	2.51
18:1 (t6+t8)	Trans 6-8-Octadecenoic acid	0.16	0.03	0.11	0.25
18:1 (t9)	Elaidic acid	0.30	0.06	0.19	0.53
18:1 (t10)	Trans 10-Octadecenoic acid	0.40	0.16	0.17	1.10
18:1 (t11)	Vaccenic acid	0.61	0.15	0.38	1.10
18:1 (c9)	Oleic acid	20.18	3.35	12.66	28.95
18:1 (c12)	Cis 12-Octadecenoic acid	0.39	0.10	0.16	0.65
18:1 (t16+c14)	Trans 16 + cis14 Octadecenoic	0.25	0.05	0.12	0.34
20:1 (c9)	Gadoleic acid	0.11	0.02	0.06	0.16
PUFA					
18:2 (t9, t12)	Linoelaidic acid	0.24	0.05	0.14	0.37
18:2 (c9, c12)	Linoleic acid	2.61	0.76	0.16	3.95

18:3 (c9, c12, c15)	α-Linolenic acid	0.44	0.09	0.10	0.61
18:2 (c9, t11)	Rumenic acid	0.30	0.08	0.06	0.47
20:3 (c8, c11, c14)	Eicosatrienoic acid	0.14	0.04	0.02	0.23
20:4 (c5, c8, c11, c14)	Arachidonic acid	0.17	0.04	0.07	0.27
20:5 (c5, c8, c11, c14, c17)	Eicosapentaenoic acid	0.05	0.01	0.02	0.09
22:4 (c7, c10, c13, c16)	Docosatetraenoic acid	0.05	0.02	0.02	0.12
22:5 (c7, c10, c13, c16, c19)	Docopentaenoic acid	0.09	0.02	0.04	0.13

[1] c = cis; t = trans; SFA = saturated fatty acids; MUFA = monounsaturated fatty acids; PUFA = polyunsaturated fatty acids.[2] P1 = 1st percentile; P99 = 99th percentile.

Table S2. Descriptive statistics for all indicators of metabolic stress (n = 297).

**Traits** [1]	**Mean**	**SD**	**P1** [2]	**P99** [2]
Body measure				
BCS, score	3.10	0.24	2.50	3.50
Ultrasound measurements				
pTAG, mg/g	69.76	10.20	50.75	94.67
PVA, mm^2	1110.03	287.66	567.88	1886.19
PVD, mm	129.71	12.98	95.87	161.26
LD, mm	148.75	13.02	117.36	178.84
Hematochemical parameters				
Hematocrit, l/l	0.31	0.03	0.24	0.36
Energy-related metabolites				
Glucose, mmol/L	4.35	0.39	3.29	5.22
Cholesterol, mmol/L	4.63	1.23	1.90	7.88
NEFA, mmol/L	0.18	0.22	0.04	1.05
BHB, mmol/L	0.56	0.26	0.27	1.30
Urea, mmol/L	6.30	1.07	4.10	8.77
Creatinine, μmol/L	81.82	6.37	71.74	99.47
Liver function/hepatic damage				
AST, U/L	96.06	24.18	68.25	166.05
GGT, U/L	25.65	10.36	13.32	75.39
BILt, μmol/L	2.65	1.80	0.68	11.05
Albumin, g/L	37.01	2.30	30.60	42.04
ALP, U/L	63.55	23.64	22.70	134.21
PON, U/mL	105.87	20.64	53.87	158.27
Oxidative stress metabolites				
ROMt, mgH2O2/100mL	12.44	3.26	5.00	20.24
AOPP, μmol/L	45.82	9.54	25.50	70.63
FRAP, μmol/L	198.42	72.83	125.28	331.55
SHp, μmol/l	376.92	48.64	266.67	510.25
Inflammation/innate immunity				
Ceruloplasmin, μmol/L	1.80	0.61	0.79	3.73
PROTt, g/L	80.71	4.93	69.06	95.07
Globulins, g/L	43.69	5.32	34.28	59.17
Haptoglobin, g/L	0.39	0.34	0.10	1.50
MPO, U/L	453.97	68.07	286.10	663.21
Minerals				
Ca, mmol/L	2.52	0.12	2.19	2.78
P, mmol/L	1.95	0.36	1.07	2.82

Mg, mmol/L	1.00	0.10	0.72	1.22
Na, mmol/L	142.21	3.04	135.26	148.12
K, mmol/L	4.10	0.42	3.07	5.06
Cl, mmol/L	101.48	3.22	92.95	109.15
Zn, μmol/L	11.29	2.79	6.37	20.96

[1] BCS = body condition score; pTAG = predicted liver triacylglycerol; PVA = portal vein area; PVD = portal vein depth; LD = liver depth; NEFA = non-esterified fatty acids; BHB = β-Hydroxybutyrate; AST = aspartate aminotransferase; GGT = γ-glutamyl transferase; BILt = total bilirubin; ALP = alkaline phosphatase; PON = paraoxonase; ROMt =total reactive oxygen metabolites; AOPP = advanced oxidation protein products; FRAP = ferric reducing antioxidant power; SHp = total thiol groups; PROTt = total proteins; MPO = myeloperoxidase. [2] P1 = 1st percentile; P99 = 99th percentile.
